# Bridging structural and functional biomarkers in functional movement disorder using network mapping

**DOI:** 10.1002/brb3.2576

**Published:** 2022-04-16

**Authors:** Petr Sojka, Matěj Slovák, Gabriela Věchetová, Robert Jech, David L. Perez, Tereza Serranová

**Affiliations:** ^1^ Department of Psychiatry, Faculty of Medicine Masaryk University and University Hospital Brno Brno Czech Republic; ^2^ Department of Neurology and Center of Clinical Neuroscience Charles University in Prague 1st Faculty of Medicine and General University Hospital Prague Czech Republic; ^3^ Functional Neurological Disorder Unit Cognitive Behavioral Neurology Division Department of Neurology Massachusetts General Hospital Harvard Medical School Boston Massachusetts USA; ^4^ Division of Neuropsychiatry Department of Psychiatry Massachusetts General Hospital Harvard Medical School Boston Massachusetts USA; ^5^ Athinoula A. Martinos Center for Biomedical Imaging Massachusetts General Hospital Harvard Medical School Charlestown Massachusetts USA

**Keywords:** functional connectivity, functional movement disorder, functional neurological disorder, MRI, salience network, temporoparietal junction

## Abstract

**Background:**

There are gaps in our neurobiological understanding of functional movement disorder (FMD).

**Objectives:**

We investigated gray matter volumetric profiles in FMD, and related findings to resting‐state functional connectivity (rsFC) profiles using Human Connectome Project data.

**Methods:**

Volumetric differences between 53 FMD patients and 50 controls were examined, as well as relationships between individual differences in FMD symptom severity and volumetric profiles. Atrophy network mapping was also used to probe whether FMD‐related structural alterations preferentially impacted brain areas with dense rsFC.

**Results:**

Compared to controls without neurological comorbidities (albeit with mild depression and anxiety as a group), the FMD cohort did not show any volumetric differences. Across patients with FMD, individual differences in symptom severity negatively correlated with right supramarginal and bilateral superior temporal gyri volumes. These findings remained significant adjusting for FMD subtype or antidepressant use, but did not remain statistically significant adjusting for depression and anxiety scores. Symptom severity‐related structural alterations mapped onto regions with dense rsFC—identifying several disease epicenters in default mode, ventral attention, and salience networks.

**Conclusions:**

This study supports that FMD is a multinetwork disorder with an important role for the temporoparietal junction and its related connectivity in the pathophysiology of this condition. More research is needed to explore the intersection of functional neurological symptoms and mood.

## Graphical abstract

This study examined relationships between individual differences in FMD symptom severity and volumetric profiles. Atrophy network mapping also probed whether FMD‐related structural alterations preferentially impacted brain areas with dense rsFC. This study supports that FMD is a multi‐network disorder with an important role for the temporoparietal junction.

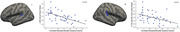

## 1 INTRODUCTION

Advances have been made in the diagnosis, treatment, and pathophysiology of functional movement disorder (FMD). (LaFaver et al., 2020, Demartini et al., 2021, Perez et al., 2021) Nonetheless, biomarkers of FMD symptom severity remain poorly understood—a factor that negatively impacts the development of biologically informed treatments. Neuroimaging studies support that FMD is associated with default mode, salience, limbic, attentional and sensorimotor network alterations—findings underscoring the importance of densely connected multimodal integration brain areas (e.g., temporoparietal junction [TPJ], cingulo‐insular areas) in the neurobiology of this condition. (Sepulcre et al., 2012) Recently, use of a dimensional, symptom severity‐informed perspective to elucidate the neural mechanisms underlying FMD has been encouraged. (Perez et al., 2021) Here, we hypothesized that FMD symptom severity and illness duration would relate to gray matter volumes in brain areas that would impact the resting‐state functional connectivity (rsFC) of densely‐connected multimodal integration brain areas. (Sepulcre et al., 2012, Fox, 2018) To test this hypothesis using a transdiagnostic approach (given that mixed symptoms are the norm rather than the exception (Butler et al., 2021), we first performed between‐group analyses to examine gray matter volumetric differences in 53 patients with a range of different FMD phenotypes versus 50 controls without neurological comorbidities. Within‐group analyses subsequently investigated relationships between individual differences in FMD symptom severity or illness duration and volumetric profiles in 50 FMD patients. Thereafter, we used Human Connectome Project (HCP) data and atrophy network mapping to identify the rsFC consequences of FMD symptom severity‐related atrophy maps.

## 2 METHODS

### 2.1 Participants

The study was approved by General University Hospital ethics committee in Prague, and all participants provided written informed consent. We enrolled 53 outpatients with nonparoxysmal FMD (42 females; age = 43.7 ± 10.1; illness duration = 5.3 ± 5.2 years) meeting clinically definite diagnostic criteria. (Gupta & Lang, 2009) Thirty‐three individuals had a range of abnormal movements (21 tremor, 11 gait difficulties, six dystonia, and four myoclonus) and 20 had isolated functional weakness. Fourteen of 33 patients with abnormal movements also had functional weakness, and two had concurrent clinically established functional seizures. Fifty controls without neurological comorbidities (36 females; age = 44.5 ± 10.0) were recruited from the community through local advertisements. Controls were included after performing a medical history and verifying a normal neurological examination. To provide a naturalistic control group that could account for common psychiatric comorbidities and medication use patterns, individuals with clinically salient depression, anxiety, and/or antidepressant use were included. Twenty‐four patients and 16 controls were on antidepressants. Exclusion criteria for all participants included age <18 years old, known magnetic resonance imaging (MRI) abnormality, intellectual disability, other major neurological/medical conditions, and psychotic/bipolar/substance use disorders.

### 2.2 Questionnaires and scales

Participants completed the Beck Depression Inventory‐II (BDI) and Spielberger State–Trait Anxiety Inventory (STAI‐trait). In patients with FMD, symptom severity was assessed using the Simplified Functional Movement Disorders Rating Scale (S‐FMDRS)—an examiner‐based rating scale with high inter‐rater reliability characterizing the presence or absence of abnormal movement in seven body regions. (Nielsen et al., 2017) Severity and duration at each body region is rated from 0 to 3 on a Likert scale; gait and speech are also rated, with a maximum total score of 54. While all participants were prospectively enrolled, 17 patients had their S‐FMDRS scores tabulated retrospectively based on a video‐recorded neurological examination performed within 2 weeks of the MRI. Fifty patients had S‐FMDRS data. See Table S1 for additional clinical score details.

### 2.3 MRI acquisition and volumetric analyses

Brain scans were acquired on a Siemens 3T Trio scanner using magnetization‐prepared rapid gradient‐echo (see Supporting Information Methods for acquisition parameters).

*FreeSurfer v7.1.1*. was used to perform cortical and subcortical reconstructions of the T1‐weighted images. Surface‐based analyses involved the removal of nonbrain tissue using a hybrid watershed algorithm, automated Talairach transformation, segmentation of subcortical white and gray matter, intensity normalization, tessellation of gray/white‐matter boundary, automated correction of topological defects, and surface deformation to form gray and white matter boundaries. Pial and gray/white matter boundary accuracy was visually inspected, and no manual corrections were needed. Vertex‐based cortical volumes were computed as surface area multiplied by thickness. A Gaussian kernel of 10 mm full‐width at half‐maximum was also applied to the subjects’ cortical volumetric maps prior to statistical analyses. Subcortical volumes were calculated using the *FreeSurfer* segmentation pipeline. All between‐ and within‐group analyses were adjusted for age, sex, and estimated total intracranial volume (eTIV).

To investigate between‐group differences, a two‐class general linear model (GLM) was used. To investigate within‐group correlations between FMD severity or illness duration and volumes, a one‐class GLM was performed. Cortical clusters were based on a 0.001 vertex‐wise threshold, and findings were subsequently corrected for multiple comparisons using Monte Carlo simulation cluster‐wise correction with 10,000 iterations and a *p*‐value < .05. In subcortical analyses, False Discovery Rate was corrected for multiple comparisons. For statistically significant findings, post hoc analyses adjusted findings for (a) depression (BDI) and trait anxiety (STAI‐trait) scores; (b) antidepressant use (yes/no); (c) FMD subtype (i.e., functional weakness yes/no).

### 2.4 Atrophy network mapping

Published methods for rsFC preprocessing steps and atrophy network mapping procedures are given in Supporting Information Methods. (Larivière et al., 2020) In brief, publicly available rsFC MRI data from an HCP healthy adult cohort (*n* = 207; 83 males; mean age ± SD = 28.7 ± 3.7 years) were used as a template to secondarily investigate whether FMD symptom severity‐related atrophy maps followed connectome organization principles (e.g., whether structural biomarkers of FMD severity preferentially mapped onto brain areas with dense rsFC profiles).

Specifically, weighted‐degree centrality was used to identify highly connected brain areas by computing the sum of all weighted connections for every region in the HCP dataset (higher weighted‐degree centrality denotes a region with greater network architecture influence). Spatial similarity between FMD symptom severity atrophy maps and centrality distributions were then compared through Pearson correlations, and statistically assessed via spatial permutation test using 10,000 repetitions.

Additionally, we identified potential “disease epicenters”—regions whose rsFC profiles spatially resembled FMD symptom severity atrophy maps. (Larivière et al., 2020) Here, FMD‐related disease epicenters were identified by spatially correlating every region's healthy rsFC profile derived from the HCP dataset to FMD symptom severity atrophy maps. This approach was repeated across the whole brain, assessing statistical significance using spatial permutation tests with 10,000 repetitions. A given brain region could be an epicenter if it is strongly connected to other high‐atrophy regions and weakly connected to low‐atrophy regions. Epicenters also do not necessarily represent the most highly connected regions (e.g., hubs) but could alternatively be closely connected to them. (Larivière et al., 2020)

## 3 RESULTS

**FIGURE 1:**
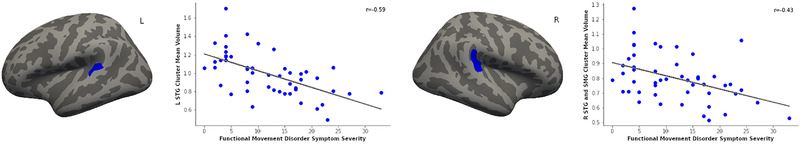
Correlations between symptom severity and gray matter volumes in 50 patients with functional movement disorder. Reduced cortical volumes in the right supramarginal gyrus and posterior aspect of the bilateral superior temporal gyri correlated with increased functional motor symptom severity. These findings were adjusted for age, sex, and estimated total intracranial volume, as well as corrected for multiple comparisons. Abbreviations: SMG, supramarginal gyrus; STG, superior temporal gyrus

Compared to controls, the FMD cohort did not show any volumetric differences. There were no statistically significant differences in symptom severity scores between patients with isolated functional weakness and other FMD phenotypes. Across FMD patients, individual differences in symptom severity negatively correlated with gray matter volumes in the right supramarginal/posterior aspect of the superior temporal gyrus (*r *= −0.43, *p*_corrected _= .0002) and posterior aspect of the left superior temporal gyrus (*r *= −0.59, *p*_corrected _= .004; Figure 1). Both clusters remained significant after adjusting for FMD subtypes or anti‐depressant use; however, these clusters did not remain significant adjusting for depression and trait anxiety scores. In a post hoc analysis, there were no statistically significant associations between TPJ gray matter volume and depression or trait anxiety scores in patients with FMD. See Figure S1 for additional information. An additional post hoc analysis examining gray matter–symptom severity relationships in only the subset of patients with functional weakness showed similar right TPJ findings (Figure S2). Across FMD patients, there were no statistically significant relationships between gray matter volumes and illness duration.

**FIGURE 2:**
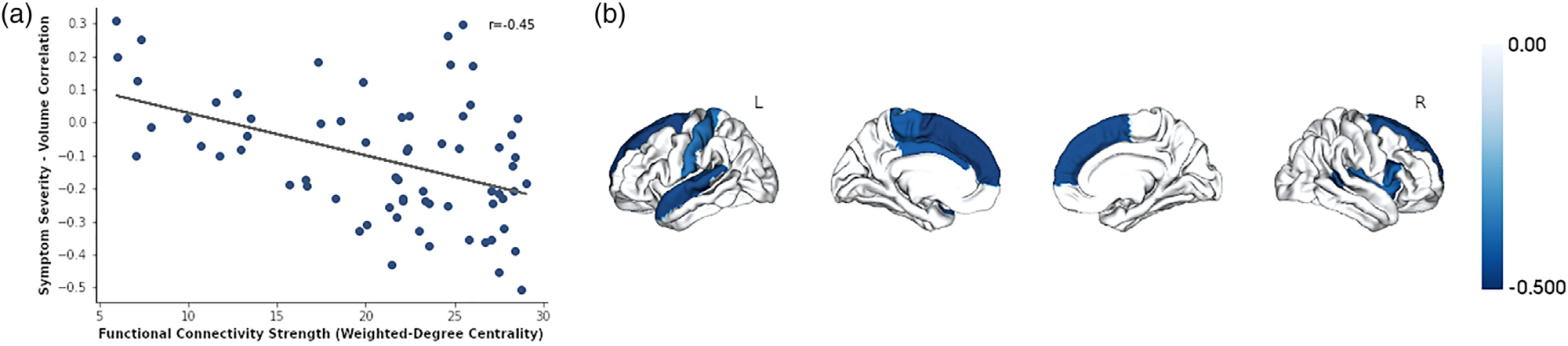
Atrophy network mapping contextualized the potential resting‐state functional connectivity relevance of identified individual differences in gray matter volumes in patients with functional movement disorder. Panel A shows that symptom severity‐related cortical atrophy spatially correlated with brain areas showing greater weighted‐degree centrality as measured using human connectome healthy subject resting‐state functional connectivity data. Panel B shows that the bilateral superior frontal and temporal gyri, right insular cortex and inferior frontal gyrus, and left middle cingulate cortex, paracentral lobule and postcentral gyrus emerged as potential disease epicenters. *Note*: The Desikan–Killiany atlas was used to parcellate cortical areas for these analyses

Spatial similarity testing revealed that brain areas displaying reduced volumes correlated with FMD symptom severity tended to be regions showing dense rsFC profiles based on HCP data (*r *= −0.45, *p*_perm_*__* = .02; Figure 2 Panel A). In patients with FMD, the bilateral superior frontal and temporal gyri, right insular cortex and inferior frontal gyrus, and left middle cingulate cortex, paracentral lobule postcentral gyrus showed disease epicenter rsFC properties (*p*_perm_*__* < .01; Figure 2 Panel B).

## 4 DISCUSSION

Here, the FMD cohort did not show any volumetric differences compared to controls. However, FMD symptom severity negatively correlated with volumetric profiles in the TPJ—specifically the right supramarginal and bilateral superior temporal gyri. Atrophy network mapping showed that these structural findings preferentially impacted higher order brain areas exhibiting increased rsFC influence (weighted‐degree centrality) based on the healthy human functional connectome.

The finding relating the TPJ to individual differences in FMD severity fits well with the neuroimaging literature implicating abnormal activity and connectivity of the TPJ in FMD. (Demartini et al., 2021, Voon et al., 2010, Maurer et al., 2016, Baek et al., 2017) The TPJ, a core node of the default mode and ventral attention networks, is an important higher order region implicated in multisensory integration, self‐agency, and stimulus‐driven attention. (Perez et al., 2021)

Our atrophy network mapping analyses help contextualize the symptom severity findings by noting that these structural alterations would be expected to impact several higher order (integrative) brain areas including the insula, middle cingulate cortex, dorsomedial prefrontal cortex, and inferior frontal gyrus. (Sepulcre et al., 2012) Given that a heterogeneous (and inconsistently identified) range of structural neuroimaging findings have been reported in FMD, we believe that it is unlikely that one definitive, microscopic structural “lesion” will be universally implicated in the pathophysiology of FMD. Rather—akin to lessons learned from lesion network mapping studies performed across a range of neuropsychiatric disorders (whereby individuals can present with disparately located structural findings that result in the *same* clinical syndrome)—it is likely that a range of structural vulnerabilities can commonly disrupt the same set of networks implicated in the pathophysiology of FMD. The findings of our study add support to the theory that FMD is a multinetwork disorder—implicating the default mode, ventral attention, and salience networks. These networks are engaged in multimodal integration, attention, prediction, interoception, and emotion processing—many of the same mechanisms involved in the neurobiology of FMD. (Baizabal‐Carvallo et al., 2019) Relatedly, a lesion network localization study of neuropsychiatric conditions with altered self‐agency (including patients with FMD) showed that a range of broadly distributed structural findings exhibited rsFC to the middle cingulate cortex. (Darby et al., 2018)

Limitations include psychiatric comorbidities, medication use, phenotypic heterogeneity, and reliance on HCP rsFC data. We did not perform a structured psychiatric interview limiting description of categorical psychiatric comorbidities. Given that patients with FMD are known to have elevates rates of depression and anxiety, we allowed controls with these mental health symptoms to be enrolled to help limit false positive between‐group findings; nonetheless, additional research is needed to further contextualize between‐group findings in patients with FMD compared to neurological and psychiatric controls matched for the severity of depression and anxiety scores. Given that phenotypic overlap is common across FMD presentations, we used a transdiagnostic approach across hyperkinetic and hypokinetic phenotypes. (Butler et al., 2021) However, whether different outward presenting phenotypes are driven by the same biological mechanisms remains to be determined. Additionally, more research is needed to investigate relationships between illness duration and volumetric profiles in patients with FMD. Lastly—the within‐group volumetric findings did not remain significant adjusting for BDI and STAI‐trait scores—suggesting that the results are at the intersection of functional motor symptoms and negative emotions. This is supported by a recent study with 152 patients with FMD that found significant correlations between S‐FMDRS and both depression and anxiety scores underscoring that both motor and nonmotor symptoms are possibly generated by the same underlying neural processes. (Forejtová et al., 2022)

In conclusion, this study supports that the default mode, ventral attention, and salience networks are important in the pathophysiology of FMD—identifying correlations between TPJ volumes and functional motor symptom severity.

## CONFLICT OF INTEREST

David L. Perez received honoraria for continuing medical education lectures in functional neurological disorder, royalties from Springer for a textbook in Functional Movement Disorder and is on the editorial board of *Epilepsy & Behavior*. The other authors declare no conflict of interest.

## FUNDING INFORMATION

Ministry of Health of the Czech Republic, Grant Number: NU20‐04‐00332 and NIMH K23MH111983 and Sidney R. Baer Jr. Foundation.

## AUTHOR CONTRIBUTIONS

*Performance of statistical analyses, data interpretation, and writing the first draft of the manuscript*: Petr Sojka. *Data collection and review/critique of the manuscript*: Matěj Slovák. *Data collection and review/critique of the manuscript*: Gabriela Věchetová. *Data collection and review/critique of manuscript*: Robert Jech. *Advising on statistical analyses, data interpretation, and review/revision/critique of manuscript*: David L. Perez. *Project conception, data collection, data interpretation, and review/revision/critique of manuscript*: Tereza Serranová.

### PEER REVIEW

The peer review history for this article is available at https://publons.com/publon/10.1002/brb3.2576

## Supporting information

Supporting Information

Supporting Information

Supporting Information

Supporting Information

## Data Availability

Anonymized data and the neuroimaging scripts will be shared with qualified researchers on request to the corresponding author following approval by the local ethics committee.

## References

[brb32576-bib-0001] LaFaver, K. , Lang, A. E. , Stone, J. , Morgante, F. , Edwards, M. , Lidstone, S. , Maurer, C. W. , Hallett, M. , Dwivedi, A. K. , & Espay, A. J. (2020). Opinions and clinical practices related to diagnosing and managing functional (psychogenic) movement disorders: Changes in the last decade. European Journal of Neurology, 27, 975–984. 10.1111/ene.14200 32153070

[brb32576-bib-0002] Demartini, B. , Nisticò, V. , Edwards, M. J. , Gambini, O. , & Priori, A. (2021). The pathophysiology of functional movement disorders. Neuroscience and Biobehavioral Reviews, 120, 387–400. 33159917 10.1016/j.neubiorev.2020.10.019

[brb32576-bib-0003] Perez, D. L. , Edwards, M. J. , Nielsen, G. , Kozlowska, K. , Hallett, M. , & LaFrance, W. C. Jr (2021). Decade of progress in motor functional neurological disorder: Continuing the momentum. Journal of Neurology, Neurosurgery, and Psychiatry, 92, 668–677. 10.1136/jnnp-2020-323953 PMC8440656 33722822

[brb32576-bib-0004] Sepulcre, J. , Sabuncu, M. R. , Yeo, T. B. , Liu, H. , & Johnson, K. A. (2012). Stepwise connectivity of the modal cortex reveals the multimodal organization of the human brain. Journal of Neuroscience, 32, 10649–10661. 10.1523/JNEUROSCI.0759-12.2012 22855814 PMC3483645

[brb32576-bib-0005] Perez, D. L. , Nicholson, T. R. , Asadi‐Pooya, A. B. I. , Butler, M. , Carson, A. J. , David, A. S. , Deeley, Q. , Diez, I. , Edwards, M. J. , Espay, A. J. , Gelauff, J. M. , Hallett, M. , Horovitz, S. G. , Jungilligens, J. , Kanaan, R. A. A. , Tijssen, M. A. J. , Kozlowska, K. , LaFaver, K. , … Aybek, S. (2021). Neuroimaging in functional neurological disorder: State of the field and research agenda. Neuroimage: Clinical, 30, 102623. 10.1016/j.nicl.2021.102623 34215138 PMC8111317

[brb32576-bib-0006] Fox, M. D. (2018). Mapping symptoms to brain networks with the human connectome. New England Journal of Medicine, 379, 2237–2245. 10.1056/NEJMra1706158 30575457

[brb32576-bib-0007] Butler, M. , Shipston‐Sharman, O. , Seynaeve, M. , Bao, J. , Pick, S. , Bradley‐Westguard, A. , Ilola, E. , Mildon, B. , Golder, D. , Rucker, J. , Stone, J. , & Nicholson, T. (2021). International online survey of 1048 individuals with functional neurological disorder. European Journal of Neurology, 28(11), 3591–3602. 34245646 10.1111/ene.15018

[brb32576-bib-0008] Gupta, A. , & Lang, A. E. (2009). Psychogenic movement disorders. Current Opinion in Neurology, 22, 430–436. 10.1097/WCO.0b013e32832dc169 19542886

[brb32576-bib-0009] Nielsen, G. , Ricciardi, L. , Meppelink, A. M. , Holt, K. , Teodoro, T. , & Edwards, M. (2017). A simplified version of the psychogenic movement disorders rating scale: The simplified functional movement disorders rating scale (S‐FMDRS). Movement Disorders Clinical Practice, 4, 710–716. 10.1002/mdc3.12475 30363505 PMC6174502

[brb32576-bib-0010] Larivière, S. , Rodríguez‐Cruces, R. , Royer, J. , Caligiuri, M. E. , Gambardella, A. , Concha, L. , Keller, S. S. , Cendes, F. , Yasuda, C. , Bonilha, L. , Gleichgerrcht, E. , Focke, N. K. , Domin, M. , von Podewills, F. , Langner, S. , Rummel, C. , Wiest, R. , Martin, P. , Kotikalapudi, R. , … Bernhardt, B. C. (2020). Network‐based atrophy modeling in the common epilepsies: A worldwide ENIGMA study. Science Advances, 6, eabc6457. 33208365 10.1126/sciadv.abc6457 PMC7673818

[brb32576-bib-0011] Voon, V. , Gallea, C. , Hattori, N. , Bruno, M. , Ekanayake, V. , & Hallett, M. (2010). The involuntary nature of conversion disorder. Neurology, 74, 223–228. 10.1212/WNL.0b013e3181ca00e9 20083798 PMC2809033

[brb32576-bib-0012] Maurer, C. W. , LaFaver, K. , Ameli, R. , Epstein, S. A. , Hallett, M. , & Horovitz, S. G. (2016). Impaired self‐agency in functional movement disorders: A resting‐state fMRI study. Neurology, 87, 564–570. 27385746 10.1212/WNL.0000000000002940 PMC4977370

[brb32576-bib-0013] Baek, K. , Doñamayor, N. , Morris, L. S. , Strelchuk, D. , Mitchell, S. , Mikheenko, Y. , Yeoh, S. Y. , Phillips, W. , Zandi, M. , Jenaway, A. , Walsh, C. , & Voon, V. (2017). Impaired awareness of motor intention in functional neurological disorder: Implications for voluntary and functional movement. Psychological Medicine, 47, 1624–1636. 10.1017/S0033291717000071 28183377 PMC5964459

[brb32576-bib-0014] Baizabal‐Carvallo, J. F. , Hallett, M. , & Jankovic, J. (2019). Pathogenesis and pathophysiology of functional (psychogenic) movement disorders. Neurobiology of Disease, 127, 32–44. 10.1016/j.nbd.2019.02.013 30798005

[brb32576-bib-0015] Darby, R. R. , Ryan Darby, R. , Joutsa, J. B. M. J. , & Fox, M. D. (2018). Lesion network localization of free will. Proceedings of the National Academy of Sciences, 115, 10792–10797. 10.1073/pnas.1814117115 PMC6196503 30275309

[brb32576-bib-0016] Forejtová, Z. , Serranová, T. , Sieger, T. , Slovák, M. , Nováková, L. , Věchetová, G. , Růžička, E. , & Edwards, M. J. (2022). The complex syndrome of functional neurological disorder. Psychological Medicine, 10.1017/S0033291721005225 34991744

