# Supplementary figures and images for: Bridging structural and functional biomarkers in functional movement disorder using network mapping
Source: Brain Behav. 2022 Apr 16;12(5):e2576. doi: 10.1002/brb3.2576 (PMC9120728; doi:10.1002/brb3.2576)

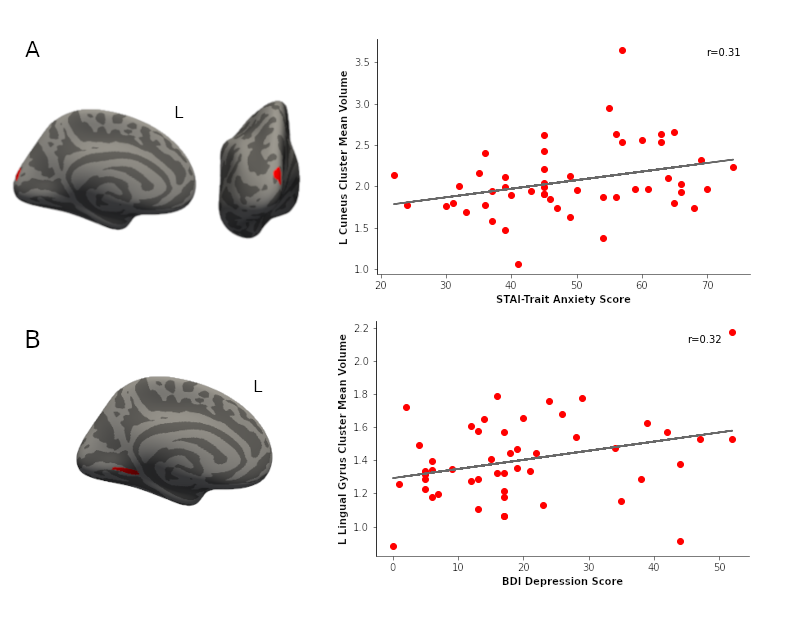

Supplement: Supplementary file 1 — Supporting Information [file BRB3-12-e2576-s002.tiff]

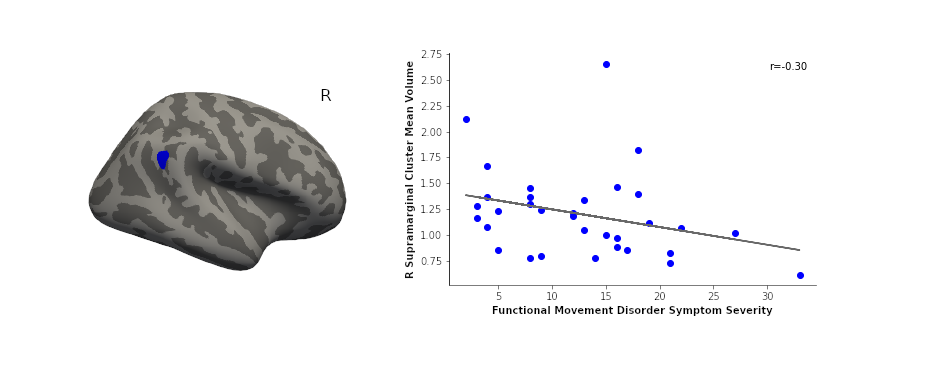

Supplement: Supplementary file 2 — Supporting Information [file BRB3-12-e2576-s004.tiff]
