# Supplementary material for: Bridging structural and functional biomarkers in functional movement disorder using network mapping
Source: Brain Behav. 2022 Apr 16;12(5):e2576. doi: 10.1002/brb3.2576 (PMC9120728; doi:10.1002/brb3.2576)
Supplement: Supplementary file 3 — Supporting Information [file BRB3-12-e2576-s001.docx]

**Supplementary Table 1.** Clinical characteristics of patients with functional movement disorder and controls.

| **Variable** | **FMD** | **Controls** | **t-statistic** | **p-value** |
| --- | --- | --- | --- | --- |
| BDI | 19.87±13.88 | 10.0±10.7 | 4.06 | 0.0001 |
| STAI-trait | 49.66±12.95 | 41.7±10.8 | 3.38 | 0.001 |
| S-FMDRS | 11.8±7.8 | - | - | - |

Data are reported as mean ± standard deviation. FMD: functional movement disorder; BDI: Beck Depression Inventory-II; STAI: Spielberger State-Trait Anxiety Inventory; S-FMDRS: Simplified Functional Movement Disorders Rating Scale.
