# Supplementary material for: Bridging structural and functional biomarkers in functional movement disorder using network mapping
Source: Brain Behav. 2022 Apr 16;12(5):e2576. doi: 10.1002/brb3.2576 (PMC9120728; doi:10.1002/brb3.2576)
Supplement: Supplementary file 4 — Supporting Information [file BRB3-12-e2576-s003.docx]

**Supplementary Methods**

**Acquisition Parameters**

The acquisition parameters for the T1-weighted images were as follows: orientation=sagittal; matrix size=256x256; voxel size=1x1x1mm; slice thickness=1mm; slices=176; repetition time=2200ms; echo time=2.43ms; field of view=230 mm.

**Resting-State Functional Connectivity (rsFC) Data Preprocessing**

rsFC data imported via Enigma toolbox^1^ were obtained from a selected group of healthy adults from the Human Connectome Project (HCP) dataset (n=207; 83 males, mean age±SD=28.73±3.73 years, range=22-36 years). The same dataset has been previously used for network-based atrophy modeling in epilepsy and only publicly available post-processed data was used for peresent set of analyses.^2^

HCP data were acquired on a Siemens Skyra 3T and included T1-weighted images using magnetization-prepared rapid gradient echo sequence with repetition time(TR)=2,400ms, echo time(TE)=2.14ms, field of view(FOV)=224✕224 mm2, voxel size=0.7✕0.7✕0.7mm, 256 slices. Resting-state blood-oxygen-level-dependent (BOLD) functional MRI used gradient-echo echo-planar imaging sequence with TR=720ms, TE=33.1ms, FOV=208✕180mm2, voxel size=2mm3, 72 slices.^1^ rsFC MRI data from the HPC dataset underwent distortion and head motion corrections, magnetic field bias correction, skull removal, intensity normalization, and were mapped to MNI152 space using previously validated methods.^1,3^ In the Enigma toolbox connectivity dataset, noise components attributed to head movement, white matter, cardiac pulsation, arterial, and large vein related contributions were automatically removed using FIX. Preprocessed time series were mapped to standard gray ordinate space using a cortical ribbon-constrained volume-to-surface mapping algorithm and subsequently concatenated to form a single time series.^1^ Functional data were parcellated according to the Desikan-Killiany brain atlas.^4^ Pairwise Pearson correlations between the time series of all cortical and subcortical (amygdala, hippocampus, thalamus, putamen, pallidum, nucleus accumbens) regions were then computed to obtain normative functional connectivity matrices. Negative connections were set to zero. Subject connectivity matrices were z-transformed and aggregated across the subjects to create a group-averaged functional connectome.^1^

**Functional Movement Disorder (FMD) Severity Related Atrophy Maps**

The FMD grey matter volume in each region of interest was obtained on the basis of Desikan-Killiany parcellation and *Freesurfer* automatic segmentation. A 1-class GLM model was used to compute symptom severity - volume correlations across 68 cortical and 14 subcortical gray matter regions. Age, sex and total intracranial volume were used as covariates of no-interest. The derived FMD severity related atrophy map was used together with normative functional connectivity data in subsequent atrophy network analyses.

**Statistical Comparison of Spatial Maps**

We used nonparametric spatial permutation tests to assess statistical significance of correspondence between rsFC and FMD severity-related atrophy maps. In cortico-cortical analyses, spatial permutation of brain maps was performed using angular permutations of spherical projections of the cortical surface.^1,5^ In subcortico-cortical analyses, we used a nonparametric null model where subcortical labels were randomly shuffled.^1^ This framework allows to control Type I error by comparing computed correlation coefficients against a null distribution determined by ensemble of correlations.

**Supplementary References**

1. [Larivière S, Paquola C, Park B-Y, Royer J, Wang Y, Benkarim O, et al. The ENIGMA Toolbox: Cross-disorder integration and multiscale neural contextualization of multisite neuroimaging datasets](http://paperpile.com/b/7WXVO8/L7ub).
2. Larivière S, Rodríguez-Cruces R, Royer J, Caligiuri ME, Gambardella A, Concha L, et al. Network-based atrophy modeling in the common epilepsies: A worldwide ENIGMA study. Sci Adv. 2020 Nov;6(47).
3. [Glasser MF, Sotiropoulos SN, Wilson JA, Coalson TS, Fischl B, Andersson JL, et al. The minimal preprocessing pipelines for the Human Connectome Project. Neuroimage. 2013 Oct 15;80:105–24.](http://paperpile.com/b/7WXVO8/mUvg)
4. [Desikan RS, Ségonne F, Fischl B, Quinn BT, Dickerson BC, Blacker D, et al. An automated labeling system for subdividing the human cerebral cortex on MRI scans into gyral based regions of interest. Neuroimage. 2006 Jul 1;31(3):968–80.](http://paperpile.com/b/7WXVO8/uLOe)
5. [Alexander-Bloch AF, Shou H, Liu S, Satterthwaite TD, Glahn DC, Shinohara RT, et al. On testing for spatial correspondence between maps of human brain structure and function. Neuroimage. 2018 Sep;178:540–51.](http://paperpile.com/b/7WXVO8/7FoI)
